# Peripheral administration of blood from tau transgenic animals exacerbates brain tau-associated pathology

**DOI:** 10.1371/journal.pone.0328470

**Published:** 2025-07-15

**Authors:** Laura Vegas-Gomez, Matias Pizarro, Jesus Garcia-Martin, Maria Angeles Arredondo-Alcala, Bianca Bustamante, Carolina Gonzalez-Silva, Soledad Matus, Rodrigo Diaz-Espinoza, Antonia Gutierrez, Rodrigo Morales, Claudia Duran-Aniotz, Ines Moreno-Gonzalez

**Affiliations:** 1 Department of Cell Biology, Genetics and Physiology, Instituto de Investigacion Biomedica de Malaga - IBIMA-Plataforma Bionand, Faculty of Sciences, Malaga University, Malaga, Spain; 2 Latin American Brain Health Institute (BrainLat), Universidad Adolfo Ibanez, Santiago, Chile; 3 Center for Social and Cognitive Neuroscience (CSCN), School of Psychology, Universidad Adolfo Ibanez, Santiago, Chile; 4 Centro Ciencia & Vida, Fundación Ciencia & Vida (FCV), Santiago, Chile; 5 Facultad de Ciencias, Universidad San Sebastián, Santiago, Chile; 6 Departamento de Biología, Facultad de Química y Biología, Universidad de Santiago de Chile,; 7 Centro de Investigacion Biomedica en Red sobre Enfermedades Neurodegenerativas (CIBERNED), Madrid, Spain; 8 Department of Neurology, The University of Texas Health Science Center at Houston, Houston, Texas, United States of America; 9 Centro Integrativo de Biologia y Quimica Aplicada (CIBQA), Universidad Bernardo O’Higgins, Santiago, Chile; Nathan S Kline Institute, UNITED STATES OF AMERICA

## Abstract

The accumulation of amyloid plaques and neurofibrillary tangles are pathological hallmarks of Alzheimer’s disease (AD). While amyloid-beta propagation through prion-like mechanisms has been extensively studied in both central and peripheral pathways, the potential spreading of tau aggregates in the periphery remains largely unexplored. Emerging evidence suggests that hyperphosphorylated tau (ptau) aggregates may propagate beyond the central nervous system, as they have been detected in peripheral tissues and biological fluids from humans and mouse models of tauopathies. However, whether peripheral ptau aggregates or other factors associated to its accumulation contribute to brain pathology remains unclear. In this study, we investigate the contribution of peripheral blood from aged P301S tau transgenic mice to tau-associated brain pathology. Blood was administered via intraperitoneal and intravenous routes to assess their effect on cognitive and motor impairment, ptau accumulation, and glial response. Our findings reveal that inoculation of blood from aged P301S mice increases tau pathology in the hippocampus, exacerbates motor and cognitive impairment, and elevates glial response. These results underscore the potential role of peripheral factors in driving brain pathology, supporting the hypothesis that blood from affected individuals contributes to the progression of tau-related neurodegeneration. Elucidating the mechanisms of tau dissemination could provide critical insights into disease progression and strengthen the rationale for targeting tau as a therapeutic strategy in AD and other tauopathies.

## Introduction

Alzheimer’s disease (AD) is the most common form of dementia in the elderly population, affecting approximately 10% of individuals over 65 years of age, with prevalence rising to nearly 40% in those over 85 years old [1,2]. With a growing elderly population and the significantly higher risk of developing AD as people age, the prevalence of this form of dementia is expected to double over the next 30 years [3]. The pathological hallmarks of AD include the extracellular accumulation of amyloid-beta (Aβ) peptides in the form of amyloid plaques and the intracellular accumulation of hyperphosphorylated tau (ptau) protein, which gives rise to neurofibrillary tangles (NFTs) [4–6]. The process of pathological aggregation and accumulation of these proteins in the brain is accompanied by a neuroinflammatory process, mediated by the activation of microglia and astroglia, as well as synaptic loss and neuronal death [7–10].

Since aggregative processes are pivotal in AD progression, this disease is considered a protein misfolding disorder (PMD), a term that includes different diseases characterized by misfolded protein deposition in specific tissues [11]. Indeed, Aβ plaques accumulation is considered one of the first pathological signs of AD, spreading through the brain via mechanisms similar to those described for infectious prions [7,8]. The amyloid cascade hypothesis proposes that this phenomenon triggers a sequence of pathological events that lead to tau aggregation, synaptic dysfunction, neuroinflammation, cell death, and cognitive decline [12–14]. Different misfolded Aβ forms are present in the AD brain, although oligomers are believed to play a key role in the initial phases of the disease [15,16]. The evaluation of Aβ aggregates as initiators of AD pathology led to a series of studies aimed at demonstrating that the presence of these aggregates has the capacity to accelerate or even induce Aβ aggregation. In this sense, it has been reported that the inoculation of brain homogenates rich in Aβ aggregates from AD patients, transgenic mice, or purified misfolded Aβ is able to induce Aβ aggregation and accelerate AD-like pathology in animal models [17–23]. We and others have shown that the administration of blood from mice displaying amyloid pathology accelerates the appearance of lesions in younger subjects. However, whether this was induced by misfolded Aβ aggregates or other factors present in the blood of donor mice is still contentious [24–27]. For instance, the pathological induction was significantly lower compared to that of mice inoculated via direct intracerebral (i.c.) injection [22,27,28]. Conversely, cyclic intravenous (i.v.) blood removal from mice expressing human APP and blood replacement using blood from WT mice, results in the amelioration of Aβ plaques and reduced brain inflammation, indicating an impact of peripheral interventions of amyloidogenic proteins in brain pathology [29].

Besides Aβ, tau pathology is another major component of AD, also exhibiting prion-like behavior and spreading along anatomically connected regions [30]. Similarly to Aβ, tau seeding and fibrillation follow a polymerization model, as do other prion-like proteins. Data suggest that tau aggregates act as pathological seeds, inducing the misfolding and aggregation of native folded tau proteins in a self-propagating manner [31,32] that can eventually spread to interconnected brain regions via synaptic transmission, vesicular transport, or perivascular routes [33,34]. Interestingly, tau aggregates are also detected in peripheral tissues such as the submandibular glands and the enteric nervous system, as well as in body fluids like the cerebrospinal fluid (CSF) and blood [33,35]. *Postmortem* analyses have identified misfolded tau in skin biopsies from AD and other tauopathy patients that exhibits seeding activity, supporting the potential of skin-derived tau as a minimally invasive biomarker with high diagnostic relevance [36]. Similarly, both Aβ and ptau aggregates have been identified in pancreatic tissue of individuals with diabetes, highlighting the importance of peripheral amyloids in disease progression [37]. Given the pathological relevance of tau, particularly its phosphorylated forms, this protein has become a key biomarker for AD diagnosis in body fluids, highlighting the potential contribution of peripheral ptau species to disease progression [38,39].

Experimental studies have confirmed that i.c. injection of tau-rich brain homogenates and preformed synthetic tau fibers induces aggregation in anatomically connected regions, supporting the notion that tau spreads along neuronal networks in a stereotypical manner [40,41]. I.c. injection of brain homogenate or paired helical filaments (PHF)-tau from AD brains in transgenic mice leads to increased plaque-associated astrogliosis, microgliosis, and enhanced Aβ deposition, suggesting a broader impact of tau pathology on neuroinflammatory response and amyloid dynamics [27,42,43]. Moreover, intraperitoneal (i.p.) administration of tau protein aggregates has provided evidence that, similar to prions and Aβ, these seeds can reach and access the central nervous system (CNS) [44]. Plasma levels of ptau are increasingly recognized as promising blood biomarkers for preclinical AD [39,45]. Evidence suggests that blood-borne tau aggregates may play an important role in triggering or initiating tau-associated pathology. It has been hypothesized that a dynamic equilibrium exists between misfolded tau species in the CNS and the periphery. Supporting this assumption, perivascular tau deposits have been observed along the blood vessels of tau transgenic mice [46], and tau present in the CSF has been shown to reach the brain parenchyma through perivascular spaces of the cerebral arteries [47]. These findings raise the possibility that tau aggregates from peripheral sources may enter the brain and modulate disease progression. However, whether ptau aggregates can reenter into the brain, and the extent to which it contributes to AD pathogenesis, remain poorly understood.

Here, we hypothesize that blood may act as a mediator facilitating the contribution of peripheral ptau to CNS pathology. Specifically, we propose that blood from aged tau- transgenic mice can exacerbate tau deposition in the brain, similarly to Aβ transmission. To investigate whether blood containing ptau aggregates can accelerate tau-associated brain pathology, we obtained peripheral blood from P301S tau transgenic mice, that exhibit extensive tau pathology, and inoculated via i.p. or i.v. into young mice. If peripheral blood components contribute to brain tau pathology, their clearance could be used as a novel therapeutic approach to ameliorate tau-related pathology in tauopathies such as AD.

## Materials and methods

### Animal model

The PS19 line is a transgenic model that encodes the human tau P301S mutation under the mouse prion protein (PrP) promoter (The Jackson Lab: Stock# 008169). The P301S model exhibits the presence of ptau deposits from 6 months old and, by 8 months, it shows the formation of neurofibrillary tangles. Microgliosis and cognitive and motor impairments are observed in these mice starting from 6 months of age [48]. WT littermate mice were used as controls. These animals were bred in the Animal Experimentation Center at the University of Málaga (CECA) under controlled conditions of temperature (21 °C ± 1 °C) and light (14 h of light and 10 h of darkness), with food and water provided *ad libitum*. All experimental procedures followed Spanish and European regulations (RD53/2013 and 2010/63/EU) and were approved by the Ethical Committee for the Use of Animals in Research at the University of Málaga (CEUMA) and the Regional Government Council (Junta de Andalucía, Spain).

### Blood collection

Male and female (1:1) 11–12 month-old tau transgenic mice and WT littermates were used as blood donors. Animals were anesthetized with sodium pentobarbital (60 mg/Kg) and sacrificed by vascular perfusion. Approximately 0.6 mL of blood per animal was collected by cardiac puncture, pooled, and inoculated into recipient mice. A solution of 0.1 M sodium citrate was used as an anticoagulant.

### Intraperitoneal and intravenous inoculation

Donor blood was injected into the peritoneal cavity in 2-month-old P301S mice (5 males and 5 females per group) using a 27G ½ tuberculin syringe coated with 50 mM sodium citrate. Three injections were performed, with each dose consisting of 150 μL of blood, once a week. Similarly, for i.v. administration, 2-month-old P301S mice (6 males and 6 females per group) were injected in the tail vein. A volume of 150 µL of donor blood was inoculated once a week for 3 weeks.

### Behavioral tests

The animals were evaluated 2 weeks before sacrificing. To study motor coordination and balance, the rotarod test was performed. This test consisted of a rotating rod with accelerating speed. The measurement of latency to fall allows the evaluation of the test performance. To assess learning and memory, animals were evaluated by Y-maze [49] and Morris water maze (MWM) [50]. The Y-maze allows the analysis of the animal’s active exploration to assess short-term working memory. Mice were placed in a maze consisting of three identical arms for 5 min, with all arms accessible for free exploration. The alternation index was analyzed. In the MWM, mice must swim through a pool in which there is a submerged platform, being introduced from different starting points on its perimeter. Mice were trained to find the platform for 4 days (4 trials/day). On day 5, the platform was removed and animals were allowed to explore the maze to determine short-term spatial memory. On day 12, animals were allowed to explore the maze without the platform to assess long-term memory. Latency and time spent in the target quadrant were recorded and compared between the groups.

### Sample collection

Animals were sacrificed at 6 months of age by vascular perfusion. Blood was collected by cardiac puncture, using 0.1 M sodium citrate as an anticoagulant. After brain removal, one half of the brain was snap-frozen at −80 °C for biochemical analyses. Frozen hemi-brains were homogenized using a lysis buffer that contains a tablet of phosphatase and protease inhibitors (A32961, Thermo Fisher Scientific) dissolved in 10 mL sterile PBS, at 10% weight/volume. The homogenate was centrifuged for 1 min at 400 × g and the supernatant was collected and frozen at −80 °C. The other half of the brain was post-fixed for 5 days at 4 °C using periodate-lysine-paraformaldehyde (PLP) in order to preserve tissue and cellular structures by crosslinking proteins and polysaccharides [51]. PLP was prepared with 4% paraformaldehyde, 75 mM lysine, 10 mM sodium metaperiodate in 0.1 M phosphate buffer (PB), pH 7.4. In i.p. injected mice, samples were dehydrated and embedded in paraffin, sliced into 10 μm thick sections with a rotating paraffin microtome and adhered onto slides. In i.v. injected animals, brains were cryoprotected in 30% sucrose, sectioned coronally at 40 μm thickness on a freezing microtome, and serially harvested in PBS and 0.02% sodium azide, for histological studies.

### ELISA

Levels of tau were measured using human-specific tau ELISA kits in plasma samples and brain homogenates. Commercial ELISA kits were used to measure the concentration of ptau at residues pS199 (KHB7041, Thermo Fisher Scientific), pT181 (KHO0631, Thermo Fisher Scientific), pT217 (MBS1608795, MyBioSource) and total human tau (KHB0041, Thermo Fisher Scientific), following manufacturer’s recommendations. Samples were read in the microplate reader FLUOstar OMEGA (BMG Biotech) at 450 nm, coupled to a computer with MARS Data Analysis software (BMG Biotech). Personnel performing these analyses were blinded to the identity of the samples.

### Western blot

Brain homogenate samples were loaded in 4–12% Bis-Tris gels (BioRad Criterion™ XT Bis-Tris Precast Gels). Proteins were transferred to nitrocellulose membranes (BioRad Trans-Blot® TurboTM) using the Trans-Blot^®^ Turbo electroblotting system (BioRad). Membranes were blocked for 1 h using 5% TBS-T BSA (Tris buffered saline, Tween 20, 5% BSA), pH 7.4. The blots were incubated with saturating amounts of the purified antibodies HT7 (Thermo Fisher Scientific; MN1000), which recognizes normal human tau, PHF-tau (residues 159 and 163), and AT8 (Thermo Fisher Scientific; MN1020), an antibody against tau species phosphorylated at S202, T205, and S208 [52]. The blots were developed using the SuperSignal™ West Pico PLUS developing solution (Thermo Fisher Scientific). Finally, they were visualized using ChemiDocTM imaging system coupled to a computer with ImageLab 6.1 software (BioRad).

### Histological analysis

Sections from experimental and control groups (4 sections/animal) were analyzed by immunohistochemistry. Antibodies against the ptau species at specific residues, PHF1, a monoclonal antibody marker (provided by Peter Davies, Albert Einstein College of Medicine, Manhasset, NY, USA) against tau species phosphorylated at pS394, and pS404 [53], and AT8 (Thermo Fisher ScientificThermo Scientific; MN1020) were used to characterize the presence of tau aggregates. Tau pathology distribution and deposition were determined. For the study of inflammation, an anti-GFAP polyclonal antibody marker (Dako; z0334) was used for astrocyte labeling, and anti-BLBP polyclonal antibody marker (Abcam; ab32423) to label reactive astrocytes. A polyclonal anti-Iba1 (Wako Chemicals; 019−19741) for labeling microglia and anti-CLEC7A monoclonal antibody (InvivoGen; mabg-mdect) to label activated microglia were used. Sections were incubated with the primary antibody, followed by the corresponding biotinylated secondary antibody (Vector Laboratories), streptavidin-conjugated horseradish peroxidase, and visualized with 0.05% 3–3-diaminobenzidine tetrahydrochloride (DAB, Sigma-Aldrich) and 0.01% hydrogen peroxide in PBS. Sections were examined under a microscope (Nikon Eclipse 80i) and images were acquired using the software Nikon ACT-2U (Nikon Corporation). Image quantification was done using ImageJ software (NIH). For double immunofluorescence, sections were sequentially incubated with the indicated primary antibodies, anti-Iba1 and anti-CLEC7A, and anti-GFAP and anti-BLBP. After primary antibody incubation, sections were washed with PBS and incubated with the corresponding Alexa 488/568 secondary antibodies (1:1000 dilution; Invitrogen). Sections were rinsed with PBS and cover-slipped with Vectashield Antifade Mounting Medium with DAPI (Vector Labs) and examined under a confocal laser microscope (Leica Stellaris 8).

### Statistical analysis

First, normality was assessed using the Shapiro-Wilk test (α = 0.05), and homoscedasticity was evaluated using Levene’s test for normally distributed data or the Fligner-Killeen test for non-normal data (α = 0.05). Group comparisons were performed with two-tailed t-test or one-way ANOVA with Tukey’s *post-hoc* tests for normally distributed data with homoscedasticity. In case equal variances could not be assumed, Welch’s t-test or Welch’s ANOVA with Games-Howell *post-hoc* tests were conducted. Non-normally distributed data was analyzed with non-parametric tests such as Mann-Whitney U test or Kruskal-Wallis test with Dunn-Bonferronni *post-hoc* test. When multiple t-tests were performed, Bonferroni-Dunn method was applied to correct for multiple comparisons. Additionally, two-way ANOVA was performed to evaluate the effect of sex on the analyzed data. All these analyses were conducted using α = 0.05. The statistical analysis and graphical representation were conducted using R (v 3.6.2) with the *rstatix* (v 0.7.2), *onewaytests* (v 3.0), *ggplot2* (v 3.4.1) and *ggprism* (v 1.0.4) packages, as well as GraphPad Prism (v 10.4.1, GraphPad Software Inc.). For the analysis of learning performance in MWM, a nonlinear mixed-effects model was employed [54,55], fitting data with Wright’s law using *nlme* package (v 3.1) in R. Comparisons of learning rates were made using t-test (α = 0.05). To analyze colocalization in double immunofluorescence images, Pearson’s correlation coefficient (r) was calculated using ImageJ software (NIH).

## Results

### Intraperitoneal inoculation of blood from P301S mice accelerates disease progression

**Fig 1:**
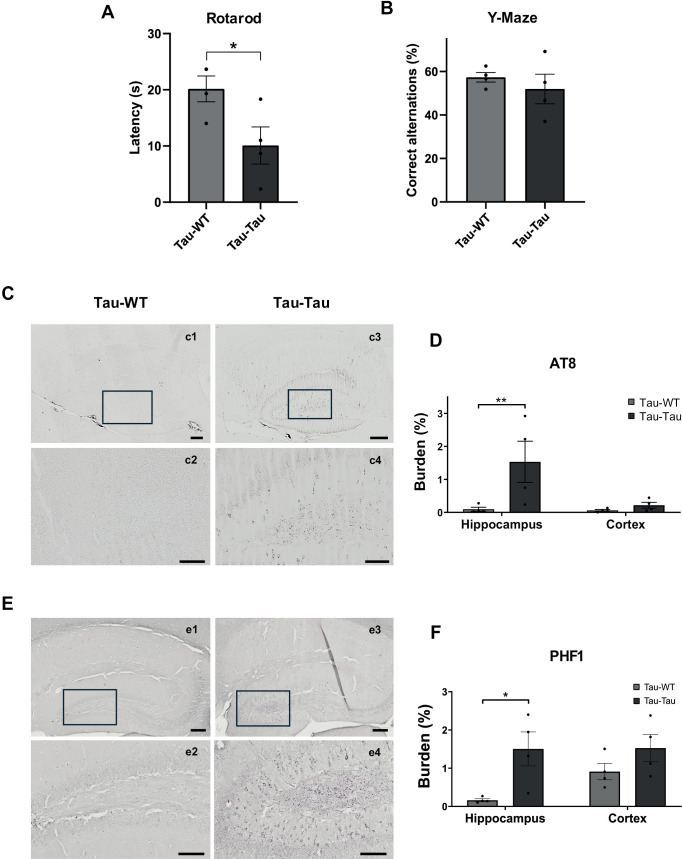
Behavioral and ptau evaluation after intraperitoneal injection of blood from aged P301S and WT mice into P301S animals. (A) The evaluation of motor activity was measured by the rotarod performance test, represented by latency to fall (s). Two tailed t-test. (B) The evaluation of working memory was measured by the Y-Maze test, represented by correct alternations (%). Two-tailed t-test. (C) Representative images of AT8 immunoreactivity in the hippocampus. Scale bars: 200 μm (c1,3), 100 μm (c2,4). (D) Quantitative analysis of burden (%) in the hippocampus and cortex. Multiple t-test were corrected using Bonferroni-Dunn method. (E) Representative images of PHF1 labeling in hippocampal sections. Scale bars: 200 μm (e1,3), 50 μm (e2,4). (F) Quantitative analysis of PHF1 burden (%) in hippocampus and cortex. Multiple t-test were corrected using Bonferroni-Dunn method. The values shown in the graphs are expressed as the mean ± SEM of the different animals used in each group (n = 4/group). *p < 0.05; **p < 0.01. Individual data points represent measurements from each animal.

To determine the effect of blood administration on disease progression, blood was obtained from donor P301S mice. At 11–12 months of age, donor animals show extensive tau intraneuronal deposits that are AT8 and PHF1-positive in the hippocampal area whereas WT donor mice did not show tau pathology (S1A and S1B Fig). Plasma isoforms pT181 and pT217 are significantly increased in aged P301S animals, in contrast with 6-month-old mice (S1C Fig). Aged P301S mice show an extensive neuroinflammation, with reactive BLBP-positive astrocytes (S1D Fig) and activated CLEC7A-positive microglia in the hippocampal area, as demonstrated by confocal colocalization (S1E Fig). Recipient P301S and WT mice were i.p. injected at 2 months of age. The animals were evaluated by different cognitive behavioral tests to measure cognitive or motor impairment associated with tauopathy. Rotarod test revealed significant impairment in motor skills when mice were infused with blood from transgenic mice compared to those injected with blood from WT animals (t_(6)_ = 2.507, p = 0.046) (Fig 1A), whereas no differences were observed in short-term working memory in the Y-maze (t_(6)_ = 0.7471, p = 0.483) (Fig 1B).

We assessed brain tau aggregation and deposition by immunohistochemistry using AT8 and PHF1 antibodies after blood i.p. administration. Statistical analysis revealed significant differences in the hippocampus (t_(12)_ = 3.195, p = 0.015 for AT8; t_(12)_ = 3.108, p = 0.018 for PHF1) but not in the cerebral cortex (t_(12)_ = 0.3470, p > 0.999 for AT8; t_(12)_ = 1.424, p = 0.360 for PHF1), showing an increased burden of tau immune-reactivity in the hippocampus of mice treated with blood from P301S transgenic mice compared to those injected with blood obtained from WT mice (Fig 1C–1F).

**Fig 2:**
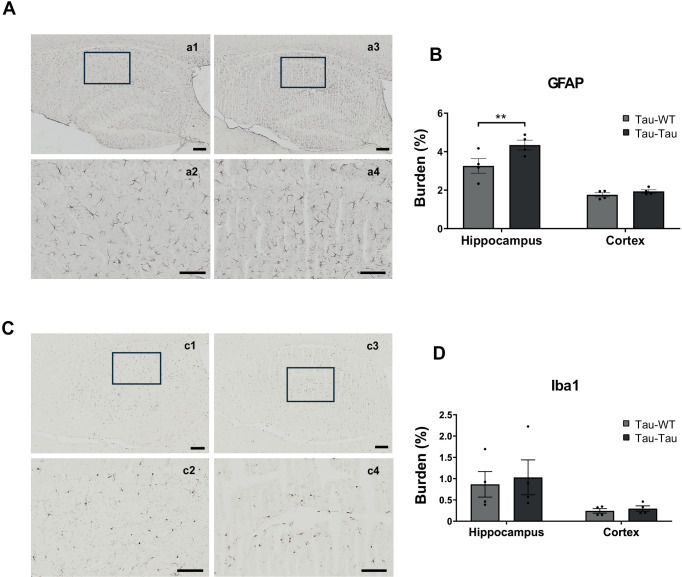
Glial response analysis after intraperitoneal infusion of blood from aged P301S and WT mice into P301S animals. (A) Representative images of GFAP labeling in hippocampal sections. Scale bars: 200 μm (a1,3), 50 μm (a2,4). (B) Quantitative analysis of GFAP burden (%) in hippocampus and cortex. Multiple t-test were corrected using Bonferroni-Dunn method. (C) Representative images of Iba1 immunoreactivity in the hippocampal area. Scale bars: 200 μm (c1,3), 50 μm (c2,4). (D) Quantitative analysis of Iba1 immuno-positive burden in the hippocampus and cortex. Multiple t-test were corrected using Bonferroni-Dunn method. The values shown in the graphs are expressed as the mean ± SEM of the different animals used in each group (n = 4/group). **p < 0.01. Individual data points represent measurements from each animal.

In addition, we assessed glial activation by identifying astrocytes (GFAP) and microglia (Iba1) in the brain of inoculated animals. The results showed a significant difference in the hippocampus in GFAP staining (t_(12)_ = 3.231, p = 0.014), but not for the cerebral cortex (t_(12)_ = 0.5332, p > 0.999) (Fig 2A and 2B). Also, no significant differences were found in the study of microgliosis between these groups in the hippocampus (t_(12)_ = 0.446, p > 0.999) nor in the cortex (t_(12)_ = 0.150, p > 0.999) (Fig 2C and 2D).

### Intravenous inoculation of P301S mice blood causes motor but not memory deficits

**Fig 3:**
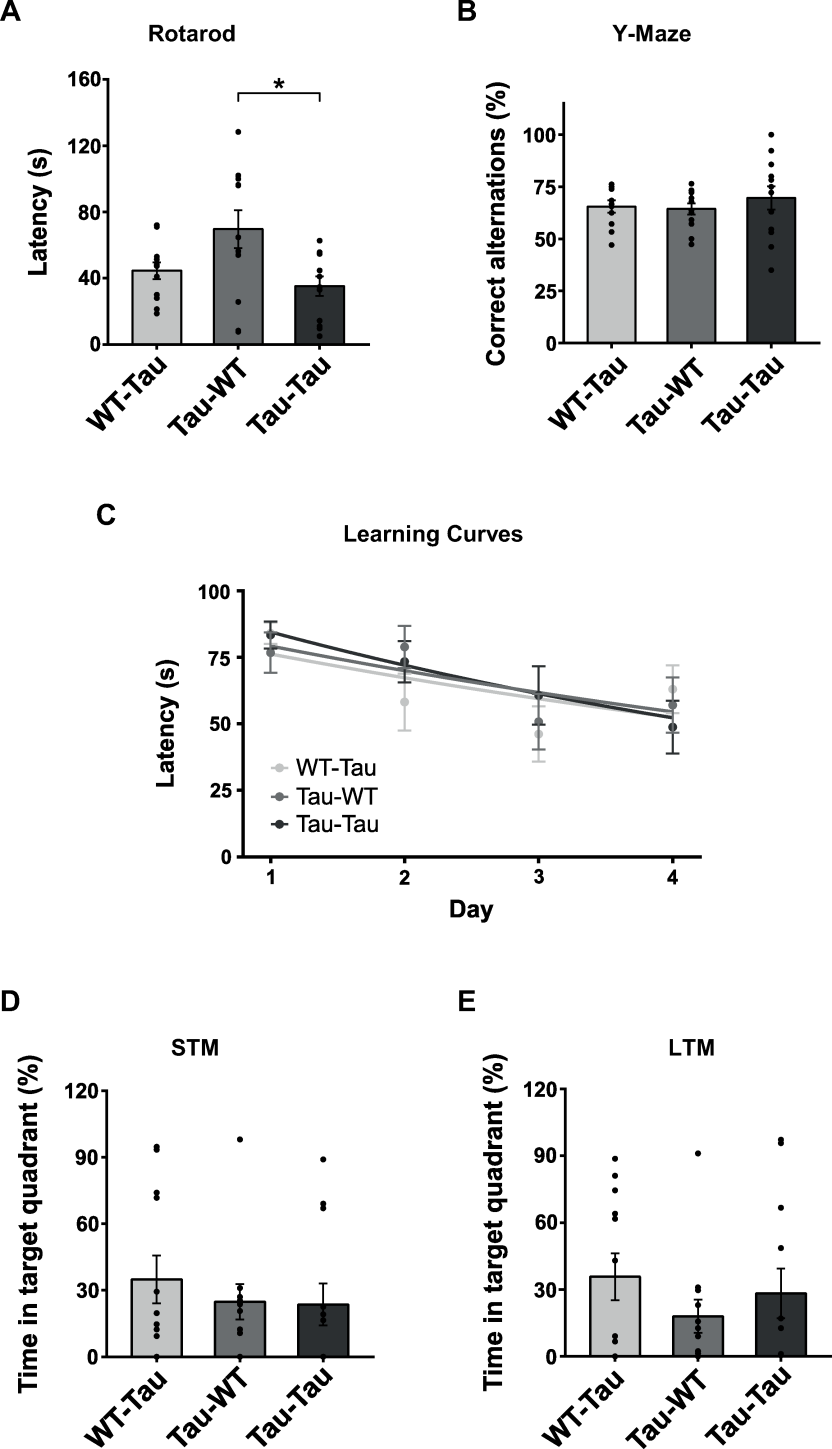
Behavioral analysis after intravenous blood infusion from old P301S and WT mice. (A) The evaluation of motor capacity was measured by the rotarod performance test, represented by latency to fall (s). Welch’s ANOVA followed by the Games-Howell multiple comparison post-hoc test. (B) The evaluation of working memory was measured by the Y-Maze test, represented by correct alternations (%). Welch’s ANOVA followed by the Games-Howell multiple comparison post-hoc test. (C) Spatial memory was evaluated with the MWM. Acquisition performance. Animals were given four trials per day and data represent the mean ± SEM of blocks of four trials. Two-tailed t-test. (D) Quantitative analysis of short-term memory on day 5 and (E) long-term memory on day 12. Kruskal-Wallis followed by the Dunn-Bonferroni multiple comparison post-hoc test. The values shown in the graphs are expressed as the mean ± SEM (n = 8-12 animals/group). *p < 0.05.

To evaluate the effect of i.v. blood inoculation, recipient P301S mice were inoculated with blood from P301S or WT donors. As a control, WT mice were i.v. inoculated with P301S blood. Mice were assessed for memory, learning, and motor function. Motor activity and coordination were evaluated using the rotarod test as P301S mice develop limb palsy over time due to the disease progression. The fall latency of the P301S blood-injected P301S mice was significantly lower than that of the WT blood-injected group (Fig 3A), as shown in *post-hoc* analysis (p = 0.042), indicating worst motor coordination after injection of blood from P301S mice. Working memory assessed using Y-maze showed no significant differences in the alternation index between groups (F_(2, 20.3)_ = 0.340, p = 0.715) (Fig 3B). Spatial memory was evaluated employing MWM. We found no changes in learning (Tau-WT, t_(103)_ = −0.380, p = 0.705, and Tau-Tau t_(103)_ = −0.002, p = 0.999) (Fig 3C). Similarly, no significant differences were observed in short-term memory (H_(2)_ = 1.530; p = 0.466) nor long-term spatial memory (H_(2)_ = 0.339, p = 0.844) between groups (Fig 3D and 3E). All analyses were controlled for potential effects of sex. No significant effects of sex or interaction were detected (S1 Table).

### Intravenous inoculation of P301S mice blood accelerates brain pathology

**Fig 4:**
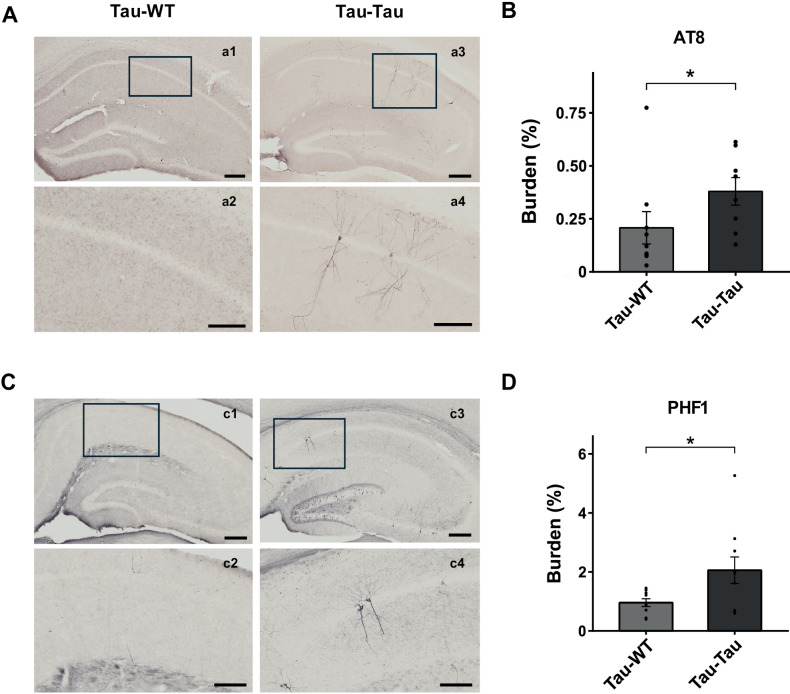
Tau histopathological analysis in P301S mice after intravenous blood infusion from P301S and WT mice. (A) Representative images of AT8 immuno-reactivity in the hippocampus. Scale bars: 200 μm (a1,3), 100 μm (a2,4). (B) Quantitative analysis of AT8 burden (%) in the hippocampal area. Mann-Whitney U test. (C) Representative images of PHF1 immuno-reactivity in the hippocampus. Scale bars: 200 μm (c1,3), 100 μm (c2,4). (D) Quantitative analysis of PHF1 burden (%) in the hippocampal area. Two-tailed t-test. The values shown in the graphs are expressed as mean ± SEM (n = 8-12 mice/group). *p < 0.05.

The progression of brain pathology was evaluated after i.v. injection of blood collected from aged P301S or WT mice. Ptau accumulation was analyzed through immunohistochemical analysis, specifically targeting pathological ptau protein in the hippocampal region using AT8 and PHF1 labeling. The results revealed a significant accumulation (W_(15)_ = 14, p = 0.036 for AT8; t_(17)_ = −2.242, p = 0.039 for PHF1) of these ptau isoforms in transgenic animals inoculated with blood from aged P301S donors, compared to those receiving blood from aged WT donors (Fig 4A–4D). No sex-related effects nor interactions were detected in any of the analyses (S1 Table).

**Fig 5:**
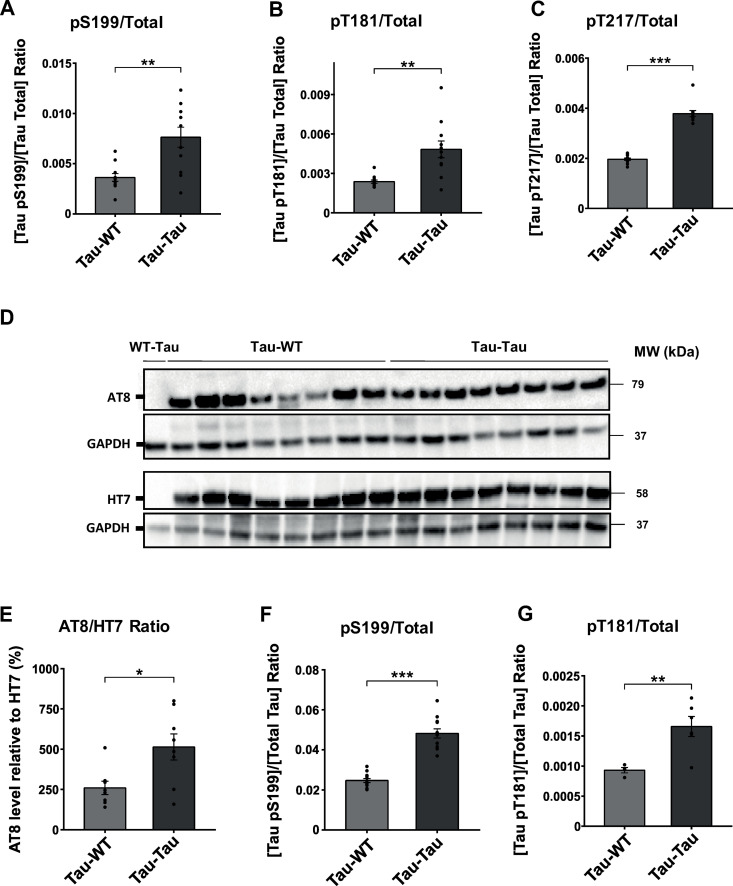
Tau isoform analysis in P301S mice after intravenous blood infusion obtained from P301S or WT mice. Quantification by ELISA of ptau pS199 (A), pT181 (B), pT217 (C), and total tau ratio in plasma samples, analyzed with Welch’s t-test and Mann-Whitney U test, respectively. (D) Representative immunoblot of ptau and total tau (AT8 and HT7) in brain homogenate samples. (E) WB quantification of AT8/HT7 ratio (%). Two tailed t-test. ELISA quantification of ptau pS199 (F), pT181 (G) and total tau ratio in the brain, analyzed with Welch’s t-test and two tailed t-test, respectively. The values shown in the graphs are expressed as mean ± SEM (n = 8-12 mice/group). *p < 0.05; **p < 0.01; ***p < 0.001.

ELISA analysis was employed to determine total and human ptau protein levels in plasma and brain homogenates of P301S mice. The ptau/total tau ratio was analyzed to determine whether pathological ptau forms (Ser199, Thr181, and Thr217 residues) were altered after total tau normalization. A significant increase was observed in the ratios of pS199 (t_(12.929)_ = −3.726, p = 0.003), pT181 (W_(20)_ = 13, p = 0.001) and pT217 (W_(21)_ = 0, p < 0.001) of phosphorylated proteins analyzed in the plasma samples compared to total tau (Fig 5A–5C) in P301S mice injected with blood from P301S animals. The relative amount of ptau compared to total tau levels was further assessed using immunoblot analysis in brain samples with the monoclonal antibodies AT8 for ptau and HT7 as a marker for total tau. The results revealed a significant accumulation of ptau species in P301S animals injected with blood from P301S mice, compared to those injected with blood from WT animals (t_(14)_ = −2.798, p = 0.014) (Fig 5D–5E). Moreover, the analysis of other pathological ptau forms in the brain showed an increase in pS199 (t_(15.124)_ = −9.457, p < 0.001) and pT181 ratios (t_(8)_ = −3.432, p = 0.009) in the experimental group (Fig 5F–5G). No significant main or interaction effects involving sex were detected across these assays (S1 Table).

**Fig 6:**
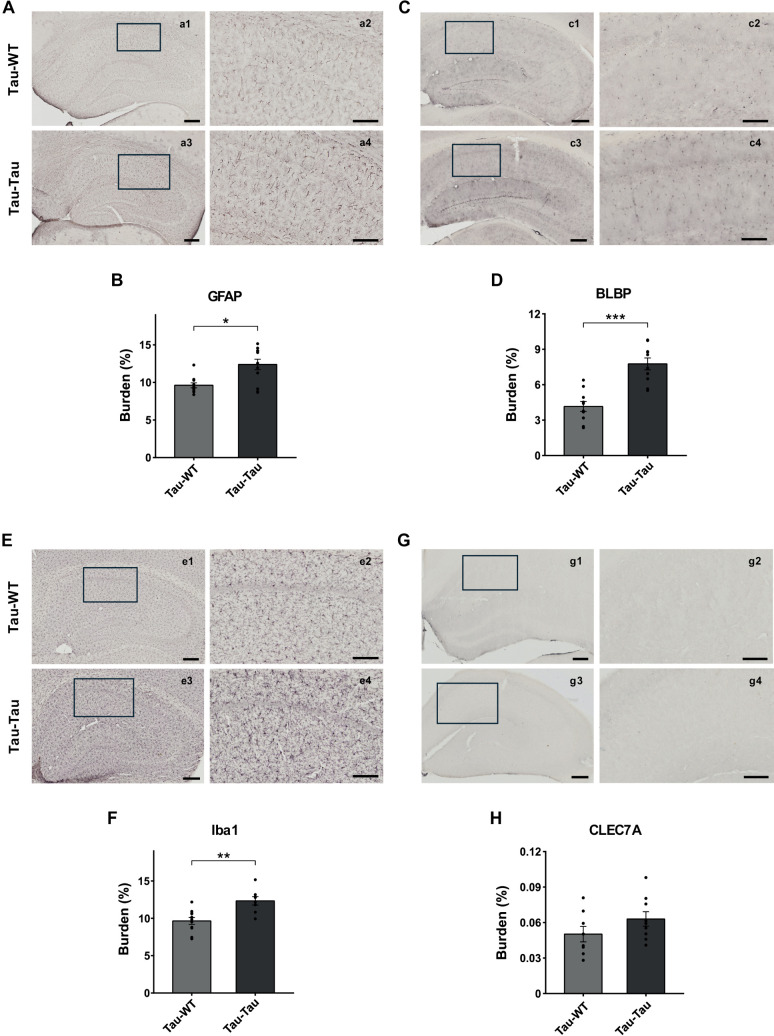
Neuroinflammation analysis after intravenous infusion of blood from P301S mice. (A) Representative images of GFAP immunostaining in the hippocampus. Scale bars: 200 μm (a1,3), 100 μm (a2,4). (B) Quantitative burden analysis of GFAP immunostaining. Mann-Whitney U test. (C) Representative images of BLBP immunoreactivity. Scale bars: 200 μm (c1,3), 100 μm (c2,4). (D) Quantitative burden analysis of BLBP immunostaining. Two-tailed t-test. (E) Representative images of Iba1 immunoreactivity in the hippocampal area. Scale bars: 200 μm (e1,3), 100 μm (e2,4). (F) Quantitative burden analysis of Iba1 immunostaining. Two-tailed t-test. (G) Representative images of CLEC7A immunolabeling. Scale bars: 200 μm (g1,3), 100 μm (g2,4). (H) Quantitative burden analysis of CLEC7A immunostaining. Two-tailed t-test. The values shown in the graphs are expressed as mean ± SEM (n = 8-12 mice/group). *p < 0.05; **p < 0.01, ***p < 0.001.

Additionally, we assessed brain inflammation by identifying astrocytes and microglia. GFAP antibody was used to detect astroglia and BLBP antibody to assess astroglial activation. Iba1 antibody was used to specifically label microglia and CLEC7A for microglial activation. The results showed both a significant increase in GFAP immuno-reactivity (W_(21)_ = 18; p = 0.019) and BLBP burden (t_(18)_ = −5.494, p < 0.001) in the hippocampus of the P301S blood injected group (Fig 6A–6D), indicating an increase in reactive astroglia. Furthermore, we also observed a significant increase in Iba1 microglial burden (t_(17)_ = −3.771, p = 0.002) in animals injected with blood from P301S mice (Fig 6E and 6F), but not significant for CLEC7A burden (t_(14)_ = −1.435, p = 0.172) (Fig 6G and 6H). The inclusion of sex as a factor did not alter the outcomes (S1 Table).

## Discussion

Here, we demonstrate that inoculation of blood from aged tau-P301S transgenic mice increases tau pathology, exacerbates motor and cognitive impairment, and elevates glial response in young tau mice. This study investigates whether blood from diseased P301S tau animals act as pathological inducers of tau accumulation through peripheral routes. We inoculated blood from P301S transgenic mice into pre-pathological P301S mice via two distinct peripheral administration routes, i.p. and i.v. Our results demonstrate that blood infusion exacerbates brain tau pathology, primarily in the hippocampus, as indicated by increased AT8 and PHF1 immunoreactivity in this area compared to controls injected with WT blood. In i.v. injected P301S mice, we also found an increase in several ptau isoforms in both blood (pS199, pT181 and pT217) and brain (pS199 and pT181), which are post-translational modifications known to be significant and correlated with disease in human patients and P301S models [56,57]. The increase of these isoforms does not exactly coincide with the most abundant human ptau forms measured in the plasma of the donor mice, indicating that the concentration of the isoform may not be a key factor of the seeding process, rather than the seeding capability of each form to facilitate the aggregation of homologous and heterologous ptau species. Further studies to determine key competent isoforms present in plasma could provide additional information about specific ptau isoforms involved in disease aggravation. Additionally, mice treated with P301S-derived blood exhibited significant motor impairments in the rotarod test after both i.p. and i.v. injection, probably due to increased degeneration of sciatic nerve fibers, as previously observed in this tauopathy model [58].

Peripherally inoculated P301S blood also exacerbates brain inflammatory response. Both i.p. and i.v. routes showed significant activation of astroglia, evidenced by increased GFAP expression, a standard marker of astrocyte reactivity [59]. Interestingly, i.v.-injected P301S mice exhibited elevated BLBP. This protein has been identified as a key regulator of the inflammatory response of astrocytes via NF-κB pathway [60,61], indicating that blood inoculation not only increases astroglial burden but it also promotes astroglial activation. Additionally, an increase in microglial response has been observed after i.v. blood administration, characterized by increased Iba1 immunoreactivity [62]. No significant increase was observed for CLEC7A-positive active microglia in recipient mice, a core activation marker enriched in disease-associated microglia subset [63,64], This may indicate that the pattern of microglia activation after injection of blood from P301S animals would be different from that seen in this disease-associated subpopulation. Furthermore, the distinct patterns of astroglial and microglial activation observed between i.p. and i.v. administration suggest that the route of peripheral exposure influences central inflammatory responses. In particular, these differences may indicate that access to vascular compartments is essential to enable tau aggregates and other blood factors to bypass peripheral clearance mechanisms and interact with the blood-brain barrier (BBB) more efficiently [65–67].

The propagation of amyloids, Aβ and tau, is a fundamental aspect of AD pathology. Aβ aggregates exhibit prion-like behavior, whereby they self-propagate and spread across neural networks via synaptic connected regions, inducing aggregation in previously unaffected areas [7,68]. Experimental studies have demonstrated that i.c. injection of Aβ aggregates into animal models accelerates amyloid formation in adjacent and anatomically connected regions, highlighting their prion-like spread within the CNS [22,69,70]. Notably, the inoculation of Aβ aggregates into peripheral routes, through i.v or i.p administration, has also been demonstrated to induce cerebral amyloid pathology, indicating that systemic Aβ seeds can cross physiological barriers and contribute to brain pathology [27,28,71]. This mechanism is likely mediated by vascular transport and the capacity of amyloid aggregates to penetrate the BBB, with aging and inflammation further amplifying these effects [65,72,73]. Similarly, studies involving the inoculation of tau-rich brain homogenates into animal models have demonstrated the spread of tau pathology, contributing to progressive neurodegeneration, mirroring the staging observed in human tauopathies [31,40,74]. Further evidence suggests that peripheral interventions can alter brain tau pathology [75–78], and peripheral removal of amyloidogenic proteins from blood can have a therapeutic effect [26,29,75]. Moreover, peripheral inflammation induction can disrupt the BBB and promote brain tau aggregation [76].

Bloodborne tau aggregates are suggested to enter the CNS via perivascular spaces, where tau seeds accumulate and act as reservoirs for ongoing pathology [65,66,79]. Perivascular tau deposition has been observed in multiple tauopathies, reinforcing the idea that vascular transport plays a crucial role in the systemic-to-CNS propagation of tau aggregates [79,80]. Multiple mechanisms may underlie the perivascular accumulation and CNS propagation of ptau forms. Although the precise processes remain unclear in humans, several tau trafficking modes have been proposed: direct cell-to-cell transmission, extracellular release and uptake of free tau, and vesicle-mediated pathways [34]. Among these, vesicle-associated transport has garnered increasing attention due to the presence of tau within exosomes and ectosomes isolated from human tauopathy brains and animal models, which have been proposed as diagnostic biomarkers [81,82]. These vesicles may facilitate ptau entry into the perivascular space and its interaction with neighboring cells. Notably, ptau can be released constitutively into the interstitial fluid (ISF) and CSF under physiological conditions, being this process modulated by neuronal activity and circadian rhythms [83]. The release is diminished in P301S transgenic mice [84], highlighting a potential disruption of ptau clearance mechanisms in disease states. Furthermore, glial cells contribute significantly to the dynamics of ptau propagation. Astrocytes and microglia are capable of internalizing tau aggregates, with microglia playing a dual role. Under normal conditions, microglial cells degrade tau aggregates and reduce pathology, but when overwhelmed, senescent, or dysfunctional, they may re-release ptau aggregates via exosomes, thereby exacerbating disease progression [85–88].

BBB dysfunction, often exacerbated by aging and neuroinflammation, may facilitate the entry of pathological proteins into the brain, further accelerating neurodegeneration [72]. Age-related deterioration of the BBB is associated with increased permeability, which allows the passage of tau aggregates, inflammatory mediators, and other harmful blood-borne factors into the brain [65,89]. Additionally, systemic inflammation and oxidative stress can impair endothelial cell function and exacerbate vascular damage, creating a permissive environment for tau aggregates to penetrate the CNS [90,91]. Aging and inflammation exacerbate the transmissibility of tau aggregates through interconnected mechanisms. Age-related changes in blood composition, including increased pro-inflammatory cytokines, have been shown to impair BBB integrity, creating a permissive environment for pathological aggregates to enter the brain [92,93]. Senescent cells further sustain this environment through the secretion of pro-inflammatory factors associated with the senescence-associated secretory phenotype. In this context, blood inoculation may introduce not only tau aggregates but also circulating pro-inflammatory factors capable of modulating the neurovascular unit. These systemic components could further compromise BBB integrity, thereby enhancing the permissiveness of the CNS environment to pathological tau entry. The presence of cytokines, oxidative stress markers, and other inflammatory mediators in the inoculated blood obtained from aged animals may act synergistically with pre-existing vascular vulnerabilities, exacerbating endothelial dysfunction and facilitating the dissemination of tau seeds across the BBB. Thus, the transmissibility of tau aggregates in experimental models may not solely reflect the properties of the aggregates themselves, but also the broader pro-inflammatory and pro-degenerative milieu conveyed through systemic circulation.

The findings presented in this study emphasize the influence of peripheral blood in the advancement of tau aggregation within the brain, being crucial for AD and other tauopathies, as peripheral tau aggregates represent a promising target for therapeutic interventions aimed at reducing tau burden in the CNS and mitigating the progression of tau-related neurodegenerative diseases. Strategies to neutralize tau aggregates in the bloodstream, such as the development of monoclonal antibodies or tau-clearing agents, could prevent their entry into the brain and the subsequent propagation of pathology, offering a novel approach to halting disease progression at its systemic origins [94–97]. In addition, the potential of blood-borne tau as a biomarker for early diagnosis represents a significant breakthrough. Recent advancements in blood-based assays, including ultrasensitive techniques, such as single molecule array and mass spectrometry, have demonstrated the ability to detect tau post-translational modifications, such as phosphorylation, acetylation, and ubiquitination, with high accuracy, providing a minimally invasive approach to monitor disease onset and progression [56,98,99]. Such biomarkers could revolutionize early diagnosis and enable timely therapeutic interventions, particularly in the preclinical stages of AD when treatment may be most effective.

Given the data outlined in this study, it is essential to reassess medical procedures such as blood transfusions for their potential involvement in inter-individual transmission of tau pathology. Future epidemiological investigations designed to directly evaluate these possibilities will be critical in determining whether such transmission occurs. Beyond the public health implications, our findings highlight the substantial role of blood that may contain tau aggregates and other cofactors, including cytokines and proteins associated with aging processes in tauopathies, including AD. These insights also provide a framework to reinterpret current and emerging tau-targeting therapies. For instance, anti-tau immunotherapies have primarily focused on neutralizing tau pathology within the CNS [100]. Our findings suggest that extending their scope to target peripheral tau aggregates may enhance their efficacy by reducing systemic sources of ptau seeding using a minimally invasive approach. Likewise, plasma exchange might lessen circulating ptau species or inflammatory mediators [101]. This raises the possibility that optimizing such procedures to deplete pathogenic tau from the bloodstream could further improve clinical outcomes. Targeting peripheral ptau-reservoirs could offer novel preventive approaches, particularly in at-risk or preclinical populations.

Overall, our study offers valuable insights into the role of blood and the effect of systemic interventions in brain disease progression. Future research should prioritize the analysis of tau aggregates propagation, their potential routes of systemic transmission, the influence of aging, inflammation, and blood-borne factors that form a complex interplay that drives neurodegeneration. Additional information underlying the specific mechanisms of ptau aggregates or other factors present in blood in accelerating brain pathology remain to be fully explored for the development of innovative therapeutic strategies aimed at mitigating the burden of AD and other tauopathies.

## Supporting information

S1 Fig Tau pathology and glial response in P301S and WT blood donor mice. (A) Representative pictures of AT8 and PHF1 immunostaining in the hippocampal area of WT (a1) and 11–12-month-old P301S (a2-3) animals used as blood donors. Scale bars: 100 μm (a1-3). (B) Representative immunoblot of ptau levels in P301S and WT using AT8 antibody. (C) Quantification by ELISA of ptau pS199, pT181, pT217, and total tau ratio in plasma samples, analyzed with two-tailed t-test. The values shown in the graphs are expressed as mean ± SEM (n = 8 mice/group). *p < 0.05; **p < 0.01. (D) Representative pictures of GFAP and BLBP immunostaining in the hippocampal area (d1-2), together with double immunofluorescence of both markers (d3-8; r = 0.29), in P301S donors. Scale bars: 100 μm (d1-2), 30 μm (d3-5), 10 μm (d6-8). (E) Representative pictures of Iba1 and CLEC7A immunostaining in the hippocampal area (e1-2), together with double immunofluorescence of both markers (e3-8; r = 0.31), in P301S donors. Scale bars: 100 μm (e1-2), 30 μm (e3-5), 10 μm (e6-8).
(TIF)

S1 Table Summary of statistical analyses assessing the influence of sex and its interaction across all experiments. Two-way ANOVA results for sex and interaction terms are summarized with F- and p-values. P-values below 0.05 are highlighted in red.
(XLSX)

S1 Raw Images Western blot raw data. Membranes were visualized using the ChemiDoc™ imaging system (Bio-Rad), connected to a computer running ImageLab 6.1 software. The intensity of the bands was measured using the gray value and mean intensity tools in FIJI (ImageJ). Background signal was subtracted from each band measurement to obtain the final intensity values used for analysis.
(PDF)
