# Supplementary figures and images for: Peripheral administration of blood from tau transgenic animals exacerbates brain tau-associated pathology
Source: PLoS One. 2025 Jul 15;20(7):e0328470. doi: 10.1371/journal.pone.0328470 (PMC12262873; doi:10.1371/journal.pone.0328470)

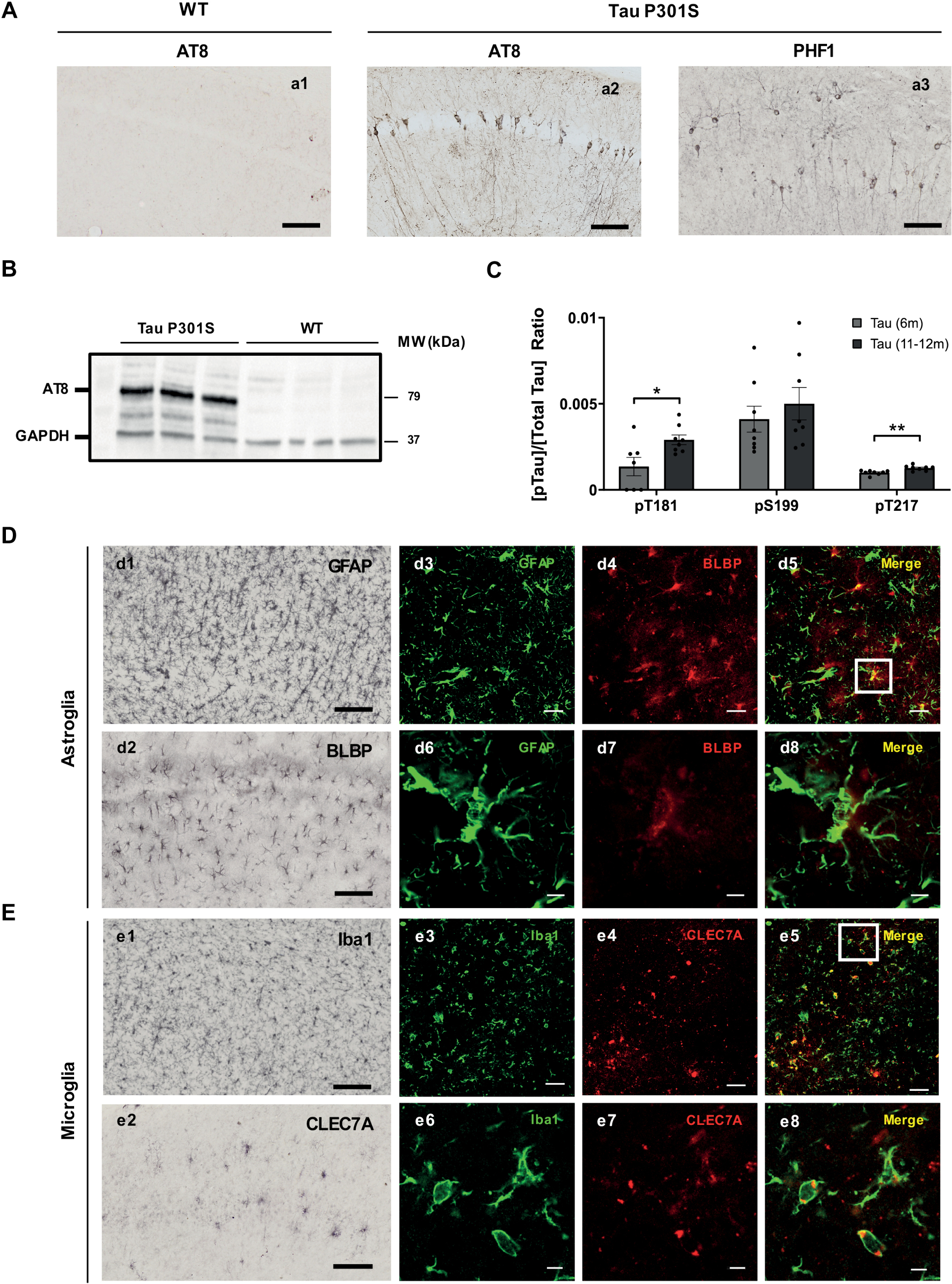

Supplement: S1 Fig — (A) Representative pictures of AT8 and PHF1 immunostaining in the hippocampal area of WT (a1) and 11–12-month-old P301S (a2-3) animals used as blood donors. Scale bars: 100 μm (a1-3). (B) Representative immunoblot of ptau levels in P301S and WT using AT8 antibody. (C) Quantification by ELISA of ptau pS199, pT181, pT217, and total tau ratio in plasma samples, analyzed with two-tailed t-test. The values shown in the graphs are expressed as mean ± SEM (n = 8 mice/group). *p < 0.05; **p < 0.01. (D) Representative pictures of GFAP and BLBP immunostaining in the hippocampal area (d1-2), together with double immunofluorescence of both markers (d3-8; r = 0.29), in P301S donors. Scale bars: 100 μm (d1-2), 30 μm (d3-5), 10 μm (d6-8). (E) Representative pictures of Iba1 and CLEC7A immunostaining in the hippocampal area (e1-2), together with double immunofluorescence of both markers (e3-8; r = 0.31), in P301S donors. Scale bars: 100 μm (e1-2), 30 μm (e3-5), 10 μm (e6-8). (TIF) [file pone.0328470.s001.tif]
