# Supplementary material for: Peripheral administration of blood from tau transgenic animals exacerbates brain tau-associated pathology
Source: PLoS One. 2025 Jul 15;20(7):e0328470. doi: 10.1371/journal.pone.0328470 (PMC12262873; doi:10.1371/journal.pone.0328470)

Fig 5D (Molecular weight marker-AT8 labelling)

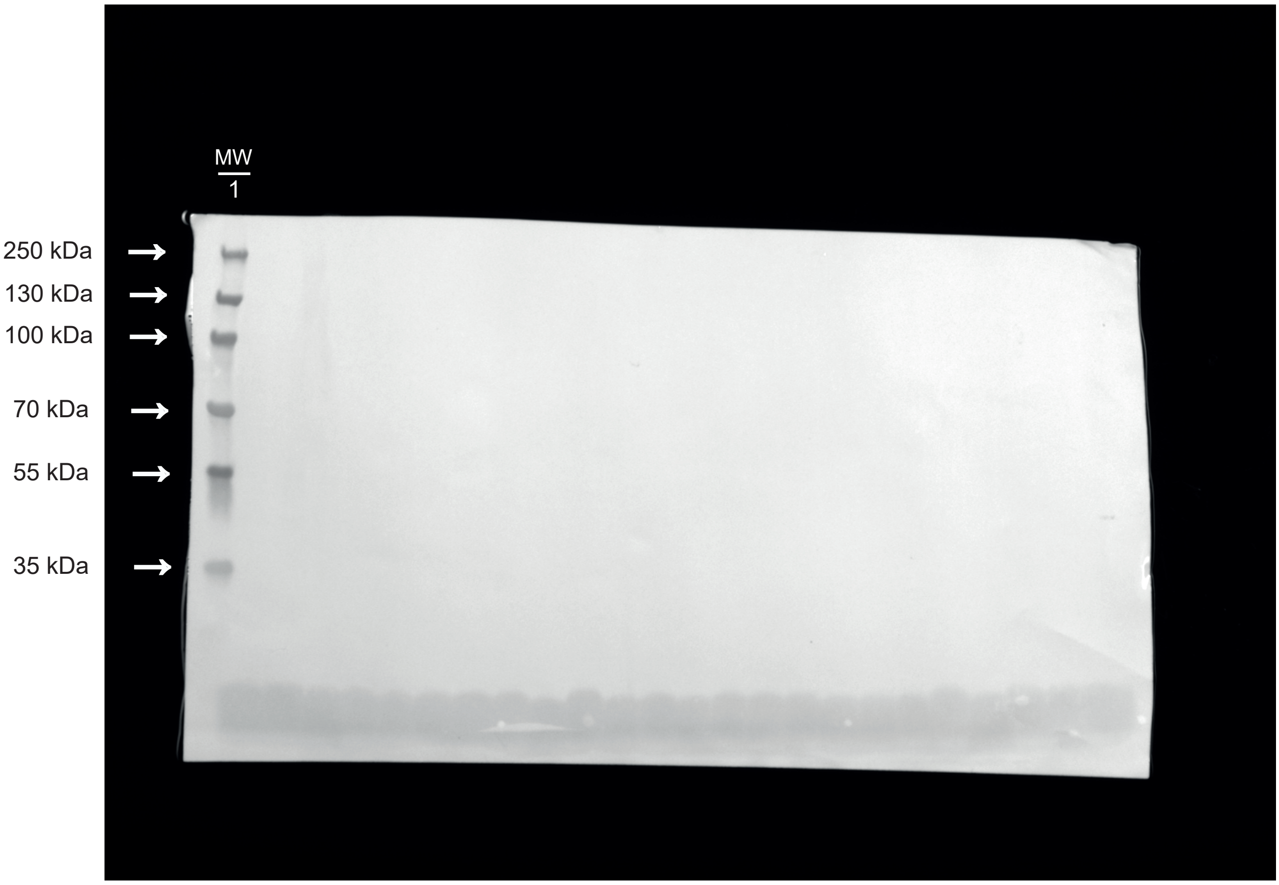

Fig 5D (AT8 labeling)

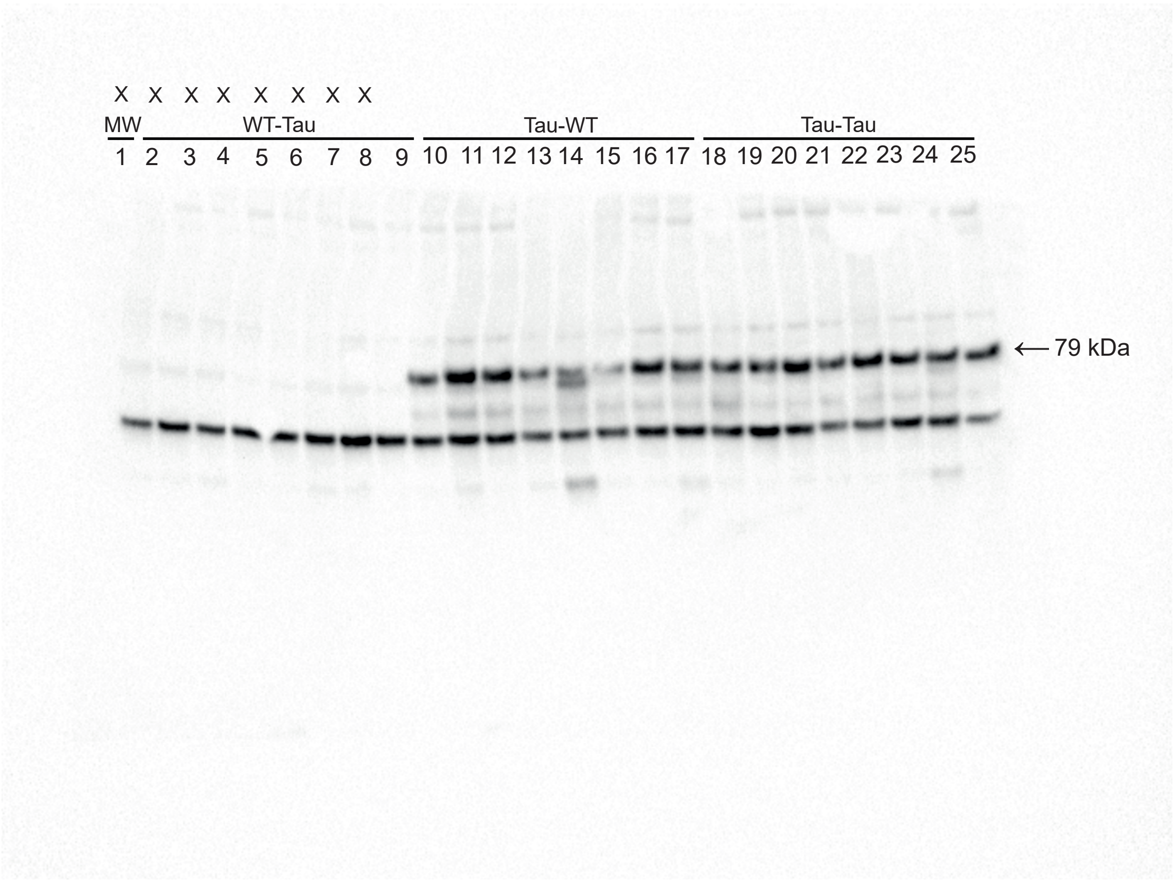

Fig 5D (GAPDH labeling-AT8 labeling)

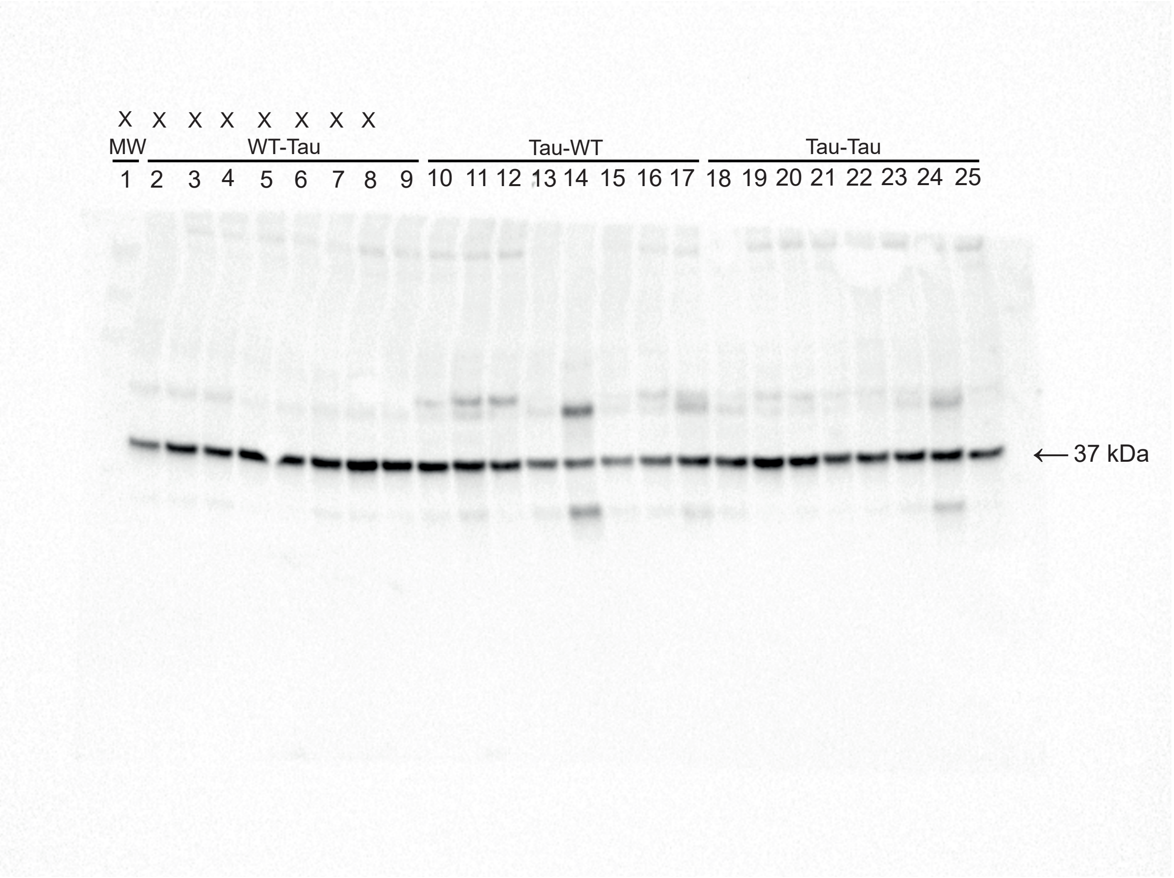

Fig 5D (Molecular weight marker-HT7 labeling)

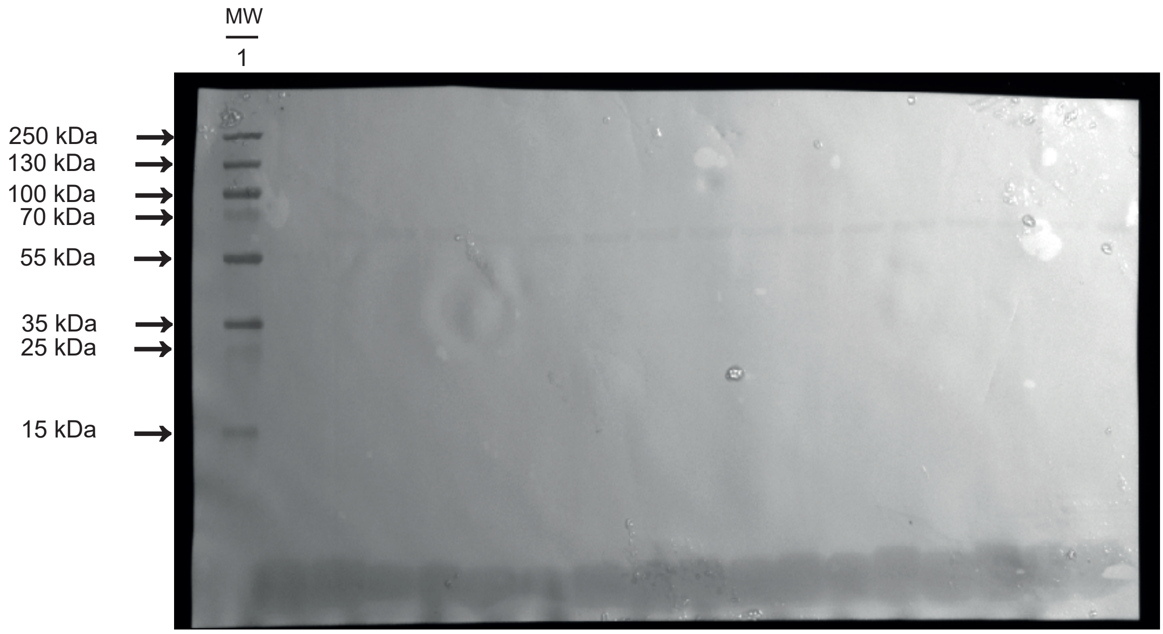

Fig 5D (HT7 labeling)

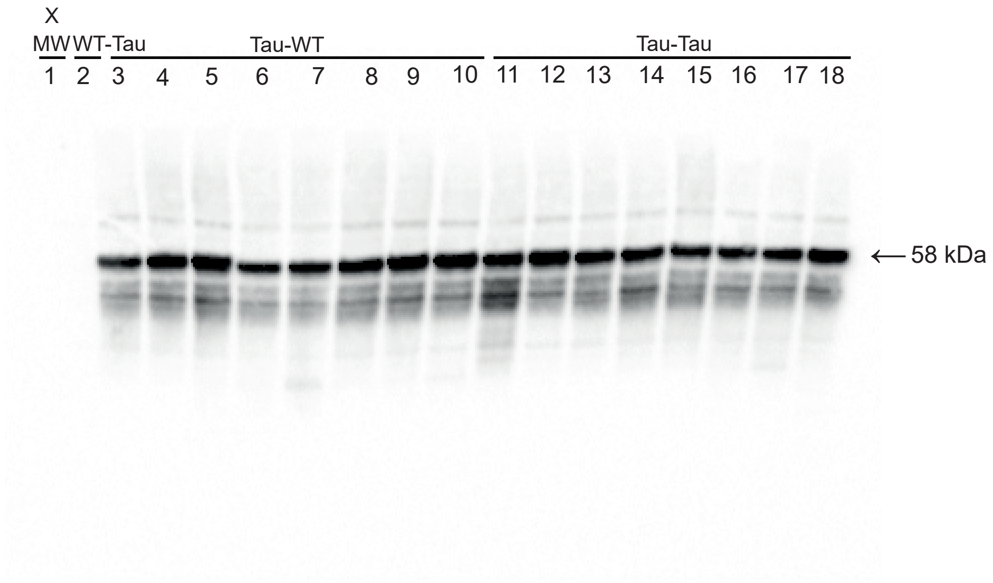

Fig 5D (GAPDH labeling-HT7 labeling)

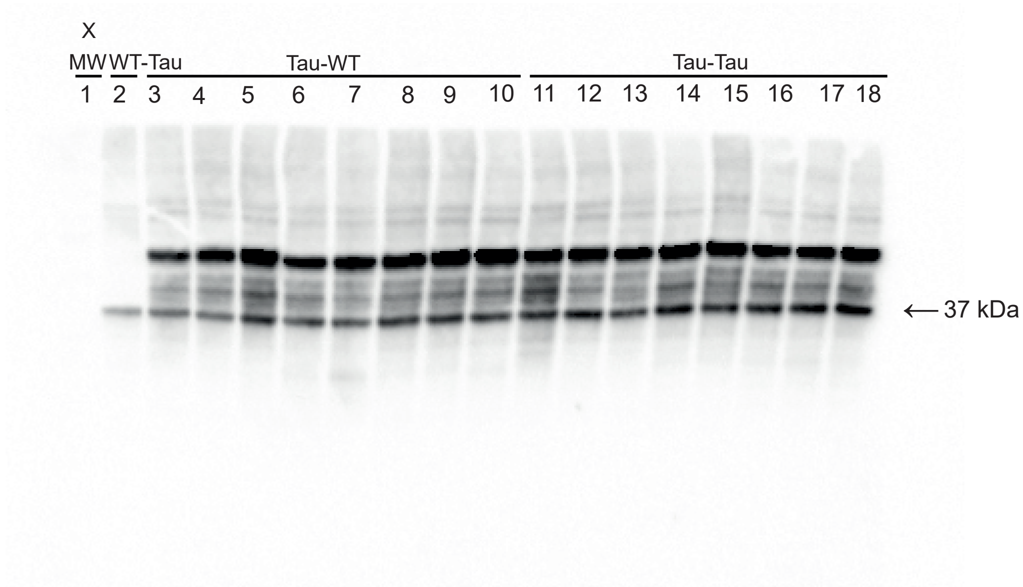

S1 Fig (Molecular weight marker)

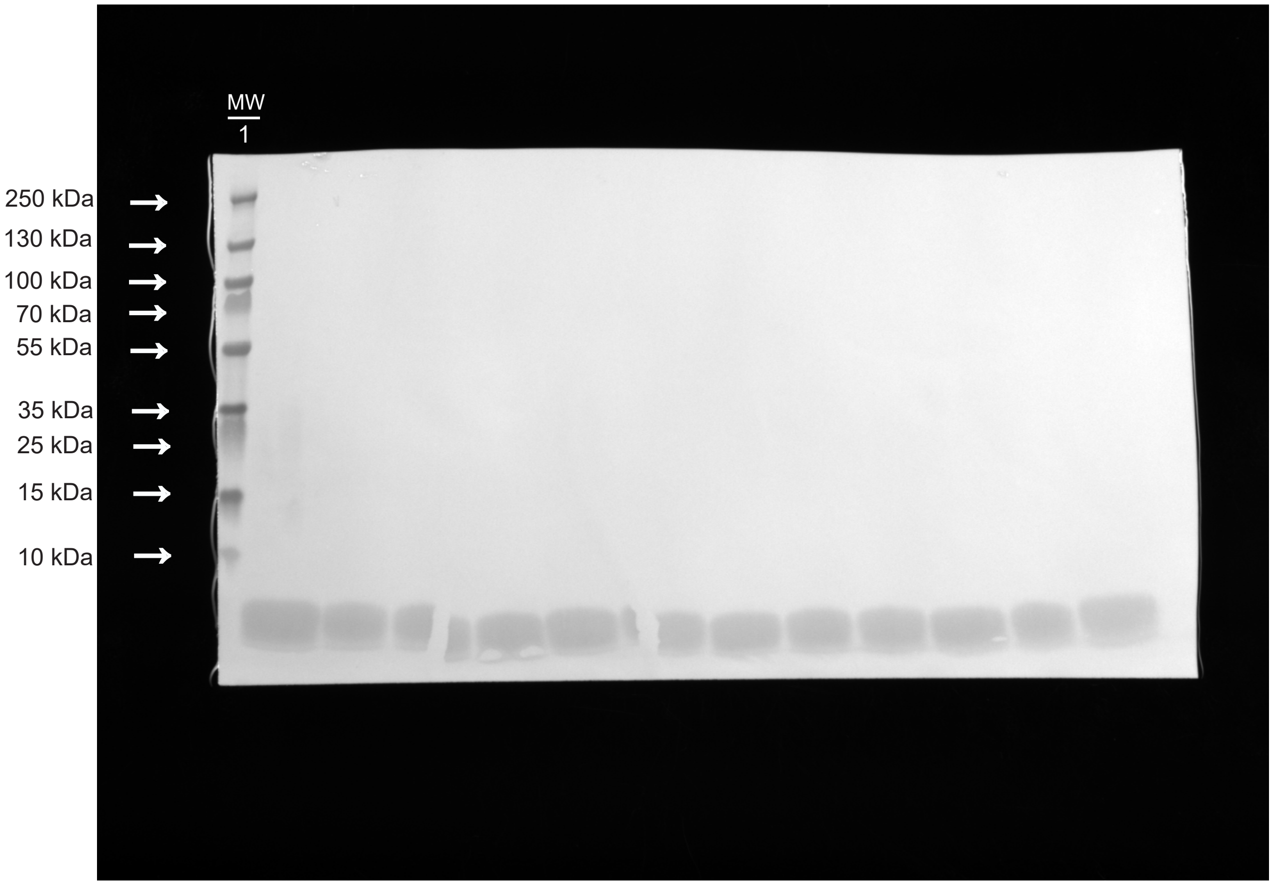

S1 Fig (AT8 and GAPDH labeling)

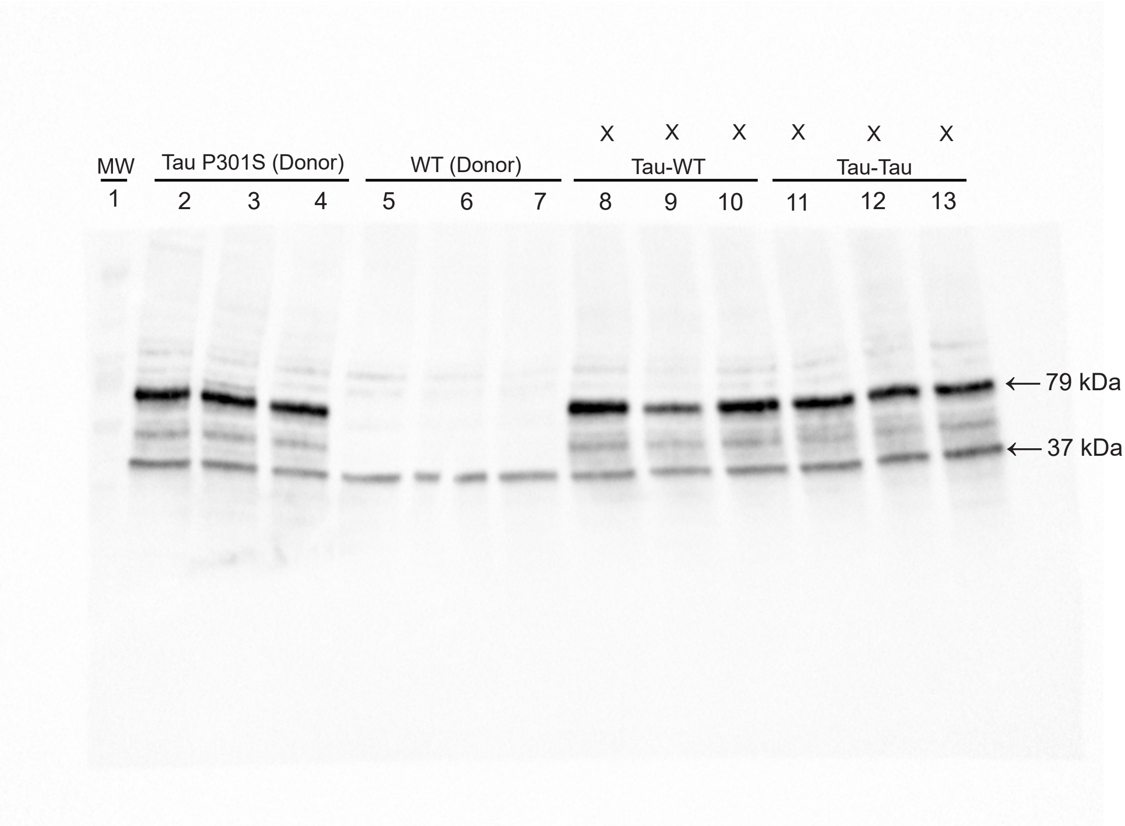

Supplement: S1 Raw Images — Membranes were visualized using the ChemiDoc™ imaging system (Bio-Rad), connected to a computer running ImageLab 6.1 software. The intensity of the bands was measured using the gray value and mean intensity tools in FIJI (ImageJ). Background signal was subtracted from each band measurement to obtain the final intensity values used for analysis. (PDF) [file pone.0328470.s003.pdf]
